# Twenty-Four-Month rhGH Intervention: Insights into Redox Regulation, Vascular Biomarkers, and Body Composition in Adult GHD Patients

**DOI:** 10.3390/ijms27031451

**Published:** 2026-01-31

**Authors:** Maria Kościuszko, Angelika Buczyńska, Justyna Hryniewicka, Agnieszka Adamska, Katarzyna Siewko, Marcin Zaniuk, Adam Jacek Krętowski, Anna Popławska-Kita

**Affiliations:** 1 Department of Endocrinology, Diabetology and Internal Medicine, Medical University of Bialystok, 15-274 Bialystok, Poland; justyna.hryniewicka@umb.edu.pl (J.H.); ak001@wp.pl (A.A.); katarzynasiewko@o2.pl (K.S.); marcin.zaniuk@gmail.com (M.Z.); adamkretowski@wp.pl (A.J.K.); annapoplawskakita@op.pl (A.P.-K.); 2 Clinical Research Center, Medical University of Bialystok, 15-274 Bialystok, Poland; angelika.buczynska@umb.edu.pl

**Keywords:** growth hormone deficiency, oxidized low-density lipoprotein, thioredoxin, vascular cell adhesion molecule 1, intercellular adhesion molecule-1, 8-oxoguanine DNA glycosylase 1

## Abstract

Adult growth hormone deficiency (GHD) is linked to increased cardiovascular and metabolic risk due to oxidative stress (OS), endothelial dysfunction, and unhealthy body composition. Long-term systemic effects of recombinant human growth hormone (rhGH) therapy remain insufficiently defined. This study assessed the impact of 24-month rhGH replacement on OS, vascular markers, body composition, and bone mineral density (BMD) in adults with severe GHD. Fifteen adults with confirmed GHD received rhGH for 24 months. Serum insulin-like growth factor 1 (IGF-1), oxidized LDL (Ox-LDL), thioredoxin (Trx), 8-oxoguanine DNA glycosylase 1 (OGG1), E-selectin, intercellular adhesion molecule 1 (ICAM-1), and vascular cell adhesion molecule 1 (VCAM-1) were measured at baseline and 12 and 24 months. Body composition and BMD were evaluated by DXA. IGF-1 increased significantly at 12 and 24 months (*p* < 0.001). Ox-LDL markedly decreased (*p* < 0.00001), while Trx and OGG1 increased (*p* < 0.05). Levels of E-selectin, ICAM-1, and VCAM-1 declined, indicating improved endothelial function. Lean body mass and BMD increased, while body fat parameters showed heterogeneous changes. Lipid profiles were unchanged. Significant correlations were observed between vascular markers and adiposity, and between BMD, triglycerides, and IGF-1. A 24-month course of rhGH therapy improves redox balance, vascular function, and body composition in adults with severe GHD, supporting the use of redox and vascular biomarkers to monitor treatment efficacy.

## 1. Introduction

Growth hormone deficiency (GHD) is a clinically recognized syndrome characterized by inadequate secretion of growth hormone (GH). In adults, it may represent the persistence of childhood-onset deficiency (CO-GHD) or arise de novo as adult-onset GHD (AO-GHD), with an estimated prevalence of approximately 2–3 cases per 10,000 individuals [1]. AO-GHD is consistently associated with increased cardiovascular risk, driven by dyslipidemia, visceral adiposity, endothelial dysfunction, and chronic low-grade inflammation [2,3,4]. Beyond these metabolic disturbances, GHD is marked by a broadened profile of vascular abnormalities that further amplify cardiovascular vulnerability. A central feature of its pathophysiology is endothelial dysfunction, reflected by reduced nitric oxide (NO) bioavailability and upregulated expression of adhesion molecules, including selectins, which collectively contribute to early atherogenic processes.

As demonstrated in our previous study and supported by existing literature, patients typically exhibit an atherogenic lipid profile—marked by elevated low-density lipoprotein cholesterol (LDL-C) and triglycerides (TGs), alongside reduced high-density lipoprotein cholesterol (HDL-C)—together with increased arterial stiffness, oxidative stress (OS), and diminished NO bioavailability [5]. These factors collectively accelerate atherogenesis [6,7,8]. Considering the central role of atherosclerosis in cardiovascular disease (CVD), heart failure, and thrombotic complications, understanding its underlying mechanisms in GHD remains essential. The interplay between oxidized low-density lipoprotein (ox-LDL), thioredoxin (Trx), vascular cell adhesion molecule 1 (VCAM-1), intercellular adhesion molecule 1 (ICAM-1), and E-selectin offers valuable mechanistic insights into atherosclerosis pathogenesis within the GHD context. Ox-LDL significantly contributes to endothelial dysfunction, inflammation, and plaque formation by reducing NO availability and increasing OS [9,10,11]. Additionally, it induces the expression of adhesion molecules, facilitating leukocyte adhesion and transmigration into the vascular intima, thereby promoting inflammation and plaque progression [12]. Trx, a major endogenous antioxidant, counteracts OS by scavenging reactive oxygen species (ROS), thereby protecting endothelial integrity and limiting oxidative damage [13,14]. Moreover, it inhibits pro-inflammatory pathways involved in atherogenesis [15]. Adhesion molecules VCAM-1, ICAM-1, and E-selectin are upregulated in response to oxidative and inflammatory stimuli, enhancing immune cell recruitment to the endothelium and aggravating vascular inflammation and remodeling [16,17,18]. Furthermore, 8-oxoguanine DNA glycosylase 1 (OGG1) plays a critical role in the base excision repair pathway by removing oxidatively damaged guanine from DNA, thus preserving genomic stability [19]. Reduced OGG1 activity has been associated with increased susceptibility to doxorubicin-induced cardiotoxicity, accelerated vascular aging, and worsened endothelial dysfunction in atherosclerosis models [20]. OGG1 deficiency leads to accumulation of oxidative DNA lesions, intensifying vascular inflammation and contributing to CVD pathogenesis [21]. Given the pivotal role of atherosclerosis in CVD, heart failure, and thrombotic events, elucidating molecular mechanisms of vascular dysfunction in GHD remains a research priority. Although recombinant human growth hormone (rhGH) therapy has demonstrated improvements in metabolic and vascular parameters, the specific contributions of Trx, OGG1, VCAM-1, ICAM-1, and E-selectin—and their interactions with lipid metabolism and body composition—have yet to be fully defined [22,23,24]. Consequently, data on endothelial and OS biomarkers in this setting remain limited.

This study aimed to evaluate the effects of 24-month rhGH therapy on cardiovascular and metabolic parameters in adults with severe GHD, with a particular focus on biomarkers of OS and endothelial dysfunction, including ox-LDL, Trx, OGG1, VCAM-1, ICAM-1, and E-selectin. By characterizing changes in these markers during long-term treatment, the research seeks to enhance understanding of the mechanisms linking GHD to increased cardiovascular risk. The findings may facilitate the integration of these biomarkers into clinical monitoring, enabling improved risk stratification and supporting personalized therapeutic strategies to reduce long-term CVD risk in this population.

## 2. Results

### 2.1. Biochemical Analysis

#### 2.1.1. IGF-1

Serum IGF-1 concentrations significantly increased after both 12 and 24 months of rhGH therapy compared with baseline values (*p* < 0.001 for both time points; Table 1).

#### 2.1.2. Ox-LDL

**Figure 1:**
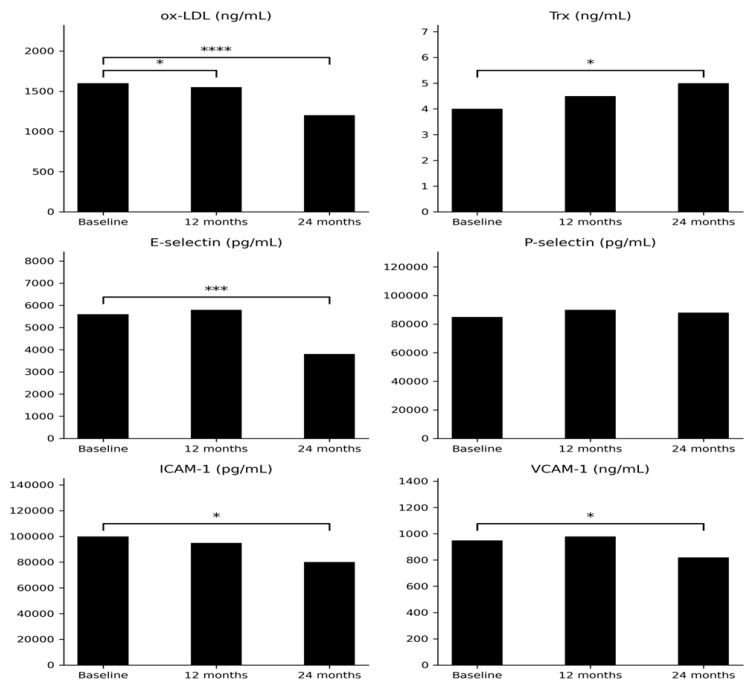
Longitudinal changes in ox-LDL, Trx, E-selectin, P-selectin, ICAM-1, and VCAM-1 during 24-month rhGH therapy at baseline, 12 months, and 24 months. Statistical significance is indicated by asterisks: * *p* < 0.05 vs. baseline; *** *p* < 0.001 vs. baseline; **** *p* < 0.00001 vs. baseline.

A significant reduction in Ox-LDL levels was observed after 12 months of rhGH therapy compared with baseline (*p* < 0.05), followed by a more pronounced decrease after 24 months (*p* < 0.00001; Table 1, Figure 1).

#### 2.1.3. Trx

Trx concentrations did not differ significantly after 12 months of therapy but were significantly higher after 24 months compared with baseline values (*p* < 0.05; Table 1, Figure 1).

#### 2.1.4. E-Selectin and P-Selectin

E-selectin concentrations significantly decreased after 24 months of rhGH therapy compared with baseline (*p* < 0.001). No statistically significant changes were observed in P-selectin levels throughout the study period (Table 1, Figure 1).

#### 2.1.5. ICAM-1 and VCAM-1

After 24 months of rhGH therapy, a statistically significant reduction in ICAM-1 concentrations was observed compared with baseline (*p* < 0.05). VCAM-1 levels also significantly decreased after 24 months (*p* = 0.05; Table 1, Figure 1).

#### 2.1.6. OGG1

A statistically significant increase in OGG1 concentration was observed after 24 months of therapy compared with baseline (*p* < 0.05; Table 1).

#### 2.1.7. Lipid Profile

No statistically significant changes were observed in total cholesterol, LDL-C, HDL-C, or TG concentrations at either 12 or 24 months of rhGH therapy compared with baseline values (Table 1).

#### 2.1.8. Vitamin D

During rhGH treatment, no statistically significant differences were observed in vitamin levels between baseline and at 12 and 24 months of therapy (Table 1).

#### 2.1.9. Glucose

Fasting glucose concentrations did not differ significantly between baseline, 12 months, and 24 months of rhGH therapy (Table 1).

##### Body Composition Evaluation

A significant increase in total mass was observed after 24 months of rhGH therapy compared with baseline (*p* = 0.05). Tissue fat (%) increased significantly after both 12 and 24 months compared with baseline (*p* = 0.04 and *p* = 0.001, respectively) while lean mass also increased, suggesting a possible change in body fat distribution, although an increase in absolute fat mass (g) was noted at 24 months (*p* = 0.05). Lean mass (g) increased significantly after 24 months of treatment (*p* = 0.0008). Significant improvements were also observed in L1–L4 BMD, femoral neck BMD, and femoral neck Z-score at 24 months compared with baseline (*p* = 0.04, *p* = 0.0002, and *p* = 0.0018, respectively). However, no statistically significant changes in BMC were detected (Table 2).

### 2.2. Correlations

#### 2.2.1. Ox-LDL and Related Correlations

A significant positive correlation was observed between ox-LDL and P-selectin after 24 months of therapy (*p* = 0.02, R = 0.60). Additionally, ox-LDL correlated positively with lean mass (g) at 12 months (*p* = 0.02, R = 0.63) (Table 3).

#### 2.2.2. Endothelial Adhesion Molecules

E-selectin demonstrated significant positive correlations with VCAM-1 (*p* = 0.05, R = 0.57) and waist circumference (*p* = 0.04, R = 0.57), as well as a negative correlation with HDL-C at 24 months (*p* = 0.01, R = −0.67). It also correlated positively with BMI (*p* = 0.02, R = 0.64) and tissue mass (g) (*p* = 0.03, R = 0.63) at 24 months, and with fat mass (g) at 12 months (*p* = 0.04, R = 0.57). VCAM-1 was positively associated with glucose concentration at 24 months (*p* = 0.03, R = 0.58). ICAM-1 levels correlated positively with BMI (*p* = 0.04, R = 0.55) and hip circumference (*p* = 0.02, R = 0.61) at 24 months, as well as with waist circumference at 18 months (*p* = 0.03, R = 0.58). A significant negative correlation with LDL-C was also observed (*p* = 0.04, R = −0.57). The above correlations are presented in Table 3.

#### 2.2.3. Redox-Related Marker

Trx showed positive correlations with tissue mass (g) and fat mass (g) (*p* = 0.04, R = 0.71; *p* = 0.01, R = 0.71, respectively), and a negative correlation with serum vitamin D levels (*p* = 0.01, R = −0.69) (Table 3).

#### 2.2.4. Lipid Profile Correlations

LDL-C demonstrated inverse correlations with the L1–L4 Z-score (*p* = 0.03, R = −0.58) and with lean mass (g) at 24 months (*p* = 0.01, R = −0.33). HDL-C correlated negatively with IGF-1 (*p* = 0.02, R = −0.59) and with lean mass (g) at both 12 months (*p* < 0.01, R = −0.73) and 24 months (*p* = 0.01, R = −0.68). Moreover, positive associations were found between TG levels and both the L1–L4 T-score (*p* = 0.03, R = 0.60) and L1–L4 Z-score (*p* = 0.05, R = 0.54) at 24 months (Table 3).

#### 2.2.5. Body Composition and Bone Parameters

Lean mass (g) showed consistent positive correlations with L1–L4 BMD, femoral neck BMD, and femoral neck T-score at both 12 and 24 months (all *p* < 0.05). At 12 months, additional correlations were observed between lean mass and the L1–L4 T-score (*p* = 0.01), L1–L4 Z-score (*p* = 0.04), and femoral neck Z-score (*p* = 0.01) (Table 3).

## 3. Discussion

In patients with GHD, numerous studies have demonstrated vascular abnormalities characteristic of early atherosclerosis, including impaired endothelium-dependent vasodilation, increased intima–media thickness (IMT), elevated arterial stiffness, and enhanced vascular inflammatory activity [25,26]. These alterations are associated with upregulated pro-inflammatory cytokines, increased expression of adhesion molecules, augmented leukocyte–endothelium interactions, and lipid abnormalities such as reduced HDL and elevated LDL levels, collectively promoting a pro-atherogenic and pro-thrombotic endothelial phenotype. In adults with GHD, rhGH replacement therapy has been shown to improve endothelial function and nitric oxide bioavailability, reduce IMT and arterial stiffness, improve lipid profiles, and attenuate vascular inflammation [25]. By evaluating a combination of traditional biochemical markers and less commonly assessed redox and vascular indicators, our findings contribute to a more integrated understanding of GH’s therapeutic impact. An early and important finding of this study was the marked rise in IGF-1 concentrations at both 12 and 24 months (*p* < 0.001), demonstrating the effective biological action of rhGH and its broad metabolic influence. IGF-1, as the main mediator of GH effects, plays a crucial role in reducing inflammation and OS by suppressing pro-inflammatory cytokines such as IL-6 and TNF-α and by boosting antioxidant defenses, including enzymes like superoxide dismutase (SOD) [27,28,29]. These protective mechanisms help maintain endothelial health and slow down the development of atherosclerosis. It is worth noting that obesity—particularly with a BMI between 30 and 35 kg/m^2^—is linked to decreased IGF-1 levels, likely due to factors such as fatty liver, increased insulin levels, and changes in IGF-binding proteins [30,31]. Conversely, some studies, including that of Andersen et al., have reported normal or even elevated IGF-1 levels in individuals with obesity [32]. In this context, given that 26.6% of our study participants were classified as obese, IGF-1 values were interpreted using a −2 SDS cutoff, in accordance with current clinical guidelines [33]. Numerous prior investigations have consistently demonstrated substantial increases in serum IGF-1 levels following rhGH therapy in adults with GHD, underscoring the biological efficacy of treatment. Bengtsson et al. reported a significant rise in IGF-1 after long-term rhGH administration, particularly among male patients, accompanied by improvements in body water and overall body composition [34]. Similarly, Christiansen et al. observed early increases in IGF-1, lean body mass, and total body water, together with reductions in fat mass after just 12 months of GH therapy (*p* < 0.001) [35]. These findings are consistent with our results, which demonstrated a significant increase in IGF-1 levels at both 12 and 24 months (*p* < 0.001), further supporting IGF-1 as a robust early biomarker of metabolic and vascular responsiveness to rhGH in adults with GHD.

Body composition analysis in our study confirmed the dual anabolic and lipolytic effects of rhGH therapy. Lean mass increased significantly (*p* = 0.0008), while body fat parameters showed heterogeneous changes, suggesting a shift in body composition rather than a uniform reduction in adiposity. Interestingly, total fat mass showed a slight but significant increase (*p* = 0.05), which may reflect a redistribution between visceral and subcutaneous compartments or compensatory adaptations in fat storage. These findings are consistent with the results of Johannsson et al. and Salomon et al. who reported improved body composition following GH replacement, although they contrast with earlier observations by Jørgensen et al. who reported reductions in total fat mass during GH therapy in adults with GHD [36,37,38]. The positive correlations observed between P-selectin and both BMI and fat mass (R = 0.64 and 0.57, respectively) further highlight the inflammatory burden imposed by adiposity, emphasizing the role of adipose tissue as an active endocrine organ contributing to vascular activation.

Moreover, BMD significantly increased at both the lumbar spine (L1–L4; *p* = 0.04) and femoral neck (*p* = 0.0002), consistent with the findings of Rahim et al. who reported enhanced bone turnover and mineralization during rhGH therapy [39]. The strong positive correlations between lean mass and BMD (e.g., R = 0.78 for L1–L4) highlight the close functional interplay between muscle and bone, particularly in GHD where anabolic signaling and mechanical loading are compromised. In contrast, BMC remained unchanged, supporting the evidence showing that early skeletal responses to GH are primarily driven by improvements in bone density and microarchitecture rather than absolute mineral content. Additionally, the positive correlations between TG and L1–L4 T- and Z-scores (R = 0.60 and 0.54) suggest a potential role of lipid availability in bone metabolism, possibly mediated by PPAR-related or adipokine-dependent pathways, as proposed by Zhou et al. [40]. The inverse associations between HDL and both IGF-1 and lean mass further underscore the complex systemic effects of GH on lipid metabolism and tissue remodeling.

A following notable finding in our study was the reduction in ox-LDL concentrations observed after 24 months of rhGH therapy (*p* < 0.00001), highlighting a potential protective effect of GH on vascular health. In our cohort, ox-LDL levels did not differ substantially between baseline and 12 months of rhGH therapy, whereas a significant reduction was observed after 24 months. This delayed response suggests that the beneficial effects of rhGH on OS may be time-dependent and require prolonged exposure. Ox-LDL reflects not only the lipid burden but also oxidative and endothelial processes, which may improve more gradually than body composition parameters. Importantly, the reduction in ox-LDL occurred despite an increase in total body mass and fat-related parameters, indicating that improvements in redox balance and lipoprotein oxidation may occur independently of changes in adiposity. Similar dissociations between OS markers and body mass have been reported in long-term hormonal or metabolic interventions, highlighting the complex and multifactorial regulation of ox-LDL. Ox-LDL is a key mediator of atherogenesis, promoting endothelial dysfunction, inflammation, and foam cell formation [41,42]. Its decline indicates an improvement in oxidative balance and reduced lipid peroxidation, consistent with previously reported GH-mediated benefits on endothelial function. Colao et al. demonstrated that long-term GH replacement in patients with GHD significantly improves endothelium-dependent vasodilation, partly through increased nitric oxide bioavailability and attenuation of oxidative stress markers [43]. Similarly, Wu et al. showed that GH and IGF-1 signaling can suppress reactive oxygen species production and vascular inflammatory responses, thereby limiting Ox-LDL–induced endothelial injury [44]. Together, these findings support the concept that rhGH therapy not only corrects hormonal deficiency but also exerts systemic antioxidant effects, potentially contributing to reduced cardiovascular risk in treated GHD patients. The observed decline in Ox-LDL levels during follow-up further supports its utility as a sensitive biomarker of vascular response to GH replacement therapy.

The increase in Trx levels observed after 24 months of rhGH therapy (*p* < 0.05), accompanied by a concurrent decrease in Ox-LDL, indicates an enhanced antioxidant activity in response to treatment. Trx is a central intracellular redox regulator that protects against oxidative injury by reducing ROS, maintaining protein thiol homeostasis, and modulating redox-sensitive signaling pathways [45]. Accordingly, the rise in Trx suggests that rhGH therapy may strengthen endogenous antioxidant defenses and counteract OS associated with GHD. This interpretation is supported by Andoh et al. who demonstrated a protective role of Trx in vascular tissue through preservation of endothelial integrity and limitation of lipid peroxidation [46]. The concomitant reduction in Ox-LDL, a key marker and mediator of atherosclerotic progression, further supports the concept that rhGH therapy mitigates vascular OS. The relationship between GH, IGF-1, and vitamin D may represent an additional modulatory pathway influencing skeletal and metabolic outcomes in adults with GHD. Vitamin D deficiency is generally associated with increased OS, endothelial dysfunction, and enhanced inflammatory activity rather than beneficial redox effects [47]. Therefore, the inverse association observed between Trx and vitamin D in the present study should be interpreted with caution. Elevated Trx concentrations at lower vitamin D levels most likely reflect a compensatory activation of endogenous antioxidant defense mechanisms in response to increased oxidative burden, rather than a protective effect of vitamin D deficiency. Similar compensatory upregulation of antioxidant systems under conditions of metabolic or inflammatory stress has been described previously [48]. Due to the observational nature of this analysis and the limited sample size, no causal conclusions can be drawn regarding the relationship between vitamin D status and redox regulation. Our findings suggest a potential role of GH in DNA repair mechanisms, as evidenced by a significant increase in OGG1 levels after 24 months of therapy (*p* < 0.05). OGG1, a key glycosylase involved in the excision of oxidized guanine lesions, is upregulated in response to OS [49]. The observed positive correlation between Ox-LDL and OGG1 supports the notion that lipid peroxidation may trigger compensatory DNA repair pathways. This aligns with data from González-Duarte et al. who reported reduced OGG1 activity in GHD patients and linked it to increased cardiovascular vulnerability [50]. Similar trends were also described by Mancini et al. who observed impaired redox-related DNA repair in untreated GHD, suggesting that GH replacement may partially restore genomic stability under oxidative conditions [51].

Despite clear improvements in redox status and endothelial markers, no significant changes were observed in traditional lipid parameters (LDL, HDL, TGs) over the course of the therapy. The interpretation of TG values should be approached with caution, as the small cohort size and the presence of individual patients with pre-existing severe hypertriglyceridemia may disproportionately influence the reported ranges. This is consistent with findings by Rudling et al. who proposed that GH modulates lipid metabolism primarily through hepatic LDL receptor expression and regulation of VLDL turnover—effects that may not be fully captured by standard fasting lipid profiles [52]. Interestingly, we observed an inverse correlation between IGF-1 and HDL levels (*p* = 0.02; R = −0.59), which contrasts with the results of Capaldo et al. who documented an increase in HDL following GH replacement [53]. Although rhGH therapy markedly increased IGF-1 concentrations, this was not accompanied by an improvement in the lipid profile. TG levels increased, while cholesterol fractions remained unchanged after 24 months. This apparent dissociation may reflect complex metabolic effects of GH and IGF-1, including enhanced lipolysis and increased hepatic TG synthesis. The observed correlations between IGF-1 and lipid parameters should therefore be interpreted with caution as they likely reflect indirect interactions between GH/IGF-1 activity, body composition, and lipid metabolism. Additionally, HDL showed a strong negative correlation with lean mass at both 12 and 24 months (*p* < 0.01; R = −0.73 and *p* = 0.01; R = −0.68, respectively), suggesting a complex, potentially bidirectional interaction between anabolic processes and lipid remodeling. This may reflect a shift in lipid distribution or function rather than concentration, emphasizing the need to interpret static lipid values within a broader metabolic context.

Endothelial function improved significantly in our study, as indicated by the reduction in circulating adhesion molecules E-selectin, ICAM-1, and VCAM-1. These molecules play key roles in leukocyte adhesion, transmigration, and the propagation of vascular inflammation, and are well-established markers of endothelial dysfunction. Their decline following rhGH therapy suggests a restoration of endothelial homeostasis and a reduction in subclinical vascular inflammation. Our results are consistent with previous reports by Christ et al. and Twickler et al. who observed time-dependent improvements in endothelial markers in adult GHD patients treated with GH [54,55]. These effects have been attributed to enhanced NO bioavailability, reduced OS, and improved insulin sensitivity—factors that collectively support endothelial repair. However, Franco et al. found no significant changes in E-selectin levels after 12 months of treatment, highlighting potential variability based on therapy duration, baseline inflammation, or individual response [56]. It is possible that improvements in endothelial biomarkers occur gradually and may only become evident with prolonged GH exposure. Additionally, early effects of GH on vascular function may be functional (e.g., NO-mediated vasodilation) rather than structural (e.g., reduced expression of adhesion molecules), which could explain the delayed changes observed in circulating markers.

Correlation analyses in our study further highlight the pro-inflammatory role of adiposity in endothelial dysfunction. E-selectin showed a positive association with waist circumference and VCAM-1, and a negative correlation with HDL (R = −0.67, *p* = 0.01), suggesting that central fat accumulation contributes to vascular inflammation and impaired lipid handling. Similarly, ICAM-1 correlated positively with BMI and waist-to-hip ratio, and negatively with LDL (R = −0.57), indicating that increased adiposity may promote chronic low-grade inflammation while altering lipid transport or clearance. These findings highlight the distinction between cross-sectional associations with adiposity-related indices and longitudinal changes induced by rhGH therapy. These findings are consistent with earlier studies by Mulhem et al. and Matsumoto et al., which identified elevated adhesion molecule levels as early indicators of cardiovascular risk in individuals with obesity or metabolic syndrome [57,58]. The observed positive correlation between VCAM-1 and fasting glucose in our cohort reinforces the link between glycemic dysregulation and endothelial stress, suggesting that metabolic disturbances may amplify vascular inflammation through upregulation of adhesion molecules. Collectively, these associations underscore the complex crosstalk between adipose tissue, lipid metabolism, and endothelial activation in GHD.

In summary, this study demonstrates that 24-month rhGH therapy in adults with severe GHD improves redox status and endothelial function and induces favorable qualitative changes in body composition, primarily reflected by increased lean mass despite heterogeneous changes in fat-related parameters. Significant reductions in Ox-LDL and adhesion molecules, together with increases in Trx and OGG1, indicate that rhGH replacement exerts antioxidant and vasculoprotective effects beyond its classical metabolic actions. Improvements in BMD and lean mass further support the anabolic and skeletal benefits of this therapy. The strengths of this study include the multidimensional assessment of biochemical, vascular, and structural parameters and the incorporation of less commonly investigated markers such as Trx and OGG1. Nevertheless, several limitations should be acknowledged. The relatively small sample size and the absence of a placebo-controlled group limit generalizability, and the observational design precludes causal inference. Variability in lipid responses may reflect unmeasured confounders, including diet, physical activity, or genetic predisposition, while the lack of detailed lipoprotein subfraction and inflammatory cytokine analyses restricts mechanistic interpretation. Despite these limitations, this work advances our knowledge by integrating vascular, redox, and genomic markers, providing new insights into the potential mechanisms underlying the long-term systemic effects of rhGH replacement. Further studies in larger cohorts with mechanistic endpoints are warranted to confirm and extend these findings.

## 4. Materials and Methods

### 4.1. Studied Population

The study was conducted at the Department of Endocrinology, Diabetology, and Internal Medicine, Medical University of Bialystok, Poland, and was supported by grant APK.002.393.2021. A total of 15 participants (4 females, 11 males), aged 18–60 years, with a confirmed diagnosis of GHD were enrolled. Severe GHD was diagnosed based on clinical features, low IGF-1 concentrations—defined as IGF-1 standard deviation scores (SDSs) below −2.0 for age and sex, in accordance with current clinical guidelines, and peak GH levels below 3 ng/mL during hypoglycemia induced by insulin and/or glucagon stimulation tests. Hormonal assessments were performed after appropriate replacement of cortisol, thyroxine, and sex steroids, as per current recommendations. Three patients were diagnosed with de novo AO-GHD, while the remaining individuals had a history of CO-GHD and had received rhGH therapy during childhood, which had been discontinued between 1 and 20 years prior to the study. Fourteen participants had multiple pituitary hormone deficiencies (MPHDs), while one patient had isolated GHD, which was confirmed by two GH stimulation tests (Table 4). The participants had no history of cardiovascular events, no current diagnosis of CVD, and were not receiving any medications known to influence cardiovascular risk (e.g., statins, ezetimibe, or antiplatelet therapy). In accordance with World Health Organization (WHO) guidelines, all the patients were advised to engage in at least 150 min of moderate-intensity physical activity per week, distributed over several days, or at least 75 min of vigorous activity, or an equivalent combination using a 2:1 ratio (e.g., 75 min of vigorous plus 150 min of moderate activity). Obese individuals received dietary counseling to follow a calorie-reduction diet targeting a daily energy deficit of 500–1000 kcal. Two male patients (aged 18 and 25 years) had type 1 diabetes, which was diagnosed 6 months before study enrollment and was treated with intensive functional insulin therapy using insulin analogs. Both achieved adequate metabolic control (glycated hemoglobin (HbA1c) < 6.5%). Exclusion criteria included poor general condition, uncontrolled diabetes (HbA1c > 7%), pre-proliferative or proliferative diabetic retinopathy, pregnancy, and any history of malignancy. All patients initiated rhGH therapy at starting doses of 0.2 mg/day (males) and 0.3 mg/day (females), with dose titration based on IGF-1 concentrations. The mean daily maintenance doses were 0.4 mg/day for men and 0.5 mg/day for women. Personalized rhGH substitution was well tolerated, and no adverse events were reported during the treatment period. Anthropometric measurements, including height and weight, were performed using standardized instruments. BMI was calculated by dividing body weight (kg) by height squared (m^2^). Waist and hip circumferences were measured using standardized procedures and were used exclusively for correlation analyses. BMD and body composition were assessed using DXA. The patients were non-smokers, did not abuse alcohol, and had no other conditions affecting peripheral markers assessment. These data were collected from medical history, physical examination, and patient records. Venous blood samples (5.5 mL) were taken after fasting, centrifuged, and the serum was stored at −80 °C. Baseline and 12-month data partially overlap with those previously published by Kościuszko et al., whereas the current study provides extended 24-month follow-up and additional analyses [5].

### 4.2. Biochemical Measurement

Serum concentrations of IGF-1, total cholesterol (CHOL), LDL-C, HDL-C, TGs, ox-LDL, Trx, OGG1, VCAM-1, ICAM-1, P-selectin, and E-selectin were assessed at baseline, after 12 months, and following completion of the 24-month rhGH therapy.

Serum IGF-1 levels were measured using the electrochemiluminescence immunoassay (ECLIA) method on a Roche Cobas e411 analyzer (Roche Diagnostics, 05061313 190; Sussex, UK) in accordance with the manufacturer’s protocol.

Lipid profile parameters, including CHOL, LDL-C, HDL-C, and TGs, were determined using the enzymatic colorimetric method on a Roche Cobas c111 analyzer (Roche Diagnostics, Basel, Switzerland) with the following reagent kits: CHOL: 03039773190; LDL-C: 03039073390; HDL-C: 03037973219; TGs: 03039773190.

OS and endothelial markers were quantified using commercially available enzyme-linked immunosorbent assay (ELISA) kits according to the manufacturers’ instructions: ox-LDL (Novus Biologicals, Bio-Techne, NBP3-18794; Minneapolis, MN, USA), Trx (XpressBio, XPEH0291; Frederick, MD, USA), OGG1 (Cloud-Clone Corp., SEC704Hu; Wuhan, China), VCAM-1 (Cloud-Clone Corp., SEA547Hu; Wuhan, China), ICAM-1 (Cloud-Clone Corp., SEA548Ca; Wuhan, China), and E-selectin (Cloud-Clone Corp., SEA029Hu; Wuhan, China).

All samples were analyzed in duplicate. Intra- and inter-assay variability were within the acceptable ranges specified by the respective manufacturers.

### 4.3. Statistical Analysis

Statistical analyses were performed using GraphPad Prism 9.0 software. Statistical significance was set at *p* < 0.05. Data distribution was assessed using the Shapiro–Wilk test and showed non-normal distribution. Accordingly, non-parametric tests were applied. Repeated-measures comparisons were performed using the Friedman test with appropriate post hoc analyses. For between-group comparisons, the Kruskal–Wallis test and the Mann–Whitney U test were used where appropriate. Spearman’s rank correlation analysis was conducted to evaluate relationships between parameters. Symbols used after the names of statistical tests indicate the type of test applied: (*) denotes the Kruskal–Wallis test, whereas (**) denotes the Mann–Whitney U test.

### 4.4. Dual-Energy X-Ray Absorptiometry and Body Composition

Body composition was assessed using DXA with a Hologic medical body analyzer (Hologic Inc., Marlborough, MA, USA). This method allows for the precise measurement of BMD, bone mineral content (BMC), body mass, total body water (TBW), fat mass, lean mass, and BMI.

## 5. Conclusions

This study confirms that 24 months of rhGH therapy in adults with severe GHD leads to sustained increases in IGF-1 levels, demonstrating effective hormonal replacement and biological responsiveness. GH therapy was associated with significant improvements in OS markers, including reductions in Ox-LDL and increases in Trx and OGG1, suggesting enhanced antioxidant capacity and activation of DNA repair pathways. Long-term treatment also resulted in decreased concentrations of endothelial adhesion molecules (E-selectin, ICAM-1, VCAM-1), indicating improved endothelial function and reduced vascular inflammation. Anabolic effects were reflected by increases in lean body mass and BMD at both the lumbar spine and femoral neck. While body fat parameters showed heterogeneous changes, total fat mass demonstrated variable responses, likely reflecting inter-individual differences in fat redistribution or metabolic adaptation. Classical lipid parameters (LDL, HDL, TGs) remained unchanged, underscoring the need for more sensitive markers to capture GH-related changes in lipid metabolism. Correlations between lipid indices, adiposity, inflammatory markers, and endothelial function highlight the complex interplay among GH action, metabolic status, and cardiovascular risk.

Collectively, these findings demonstrate the broad multisystem benefits of rhGH therapy in adult GHD and provide new insights into its potential mechanisms, particularly in redox regulation, vascular protection, and tissue remodeling.

## Figures and Tables

**Table 1:** The investigated biochemical parameters in the studied group.

Parameter					
	Initially	After 12 Months	After 24 Months	*p* Value (0 vs. 12 Months)	*p* Value (0 vs. 24 Months)
**IGF-1 (ng/mL)**	47.07 (8.57–138.8)	155.1 (36.04–265.1)	153.19 (58.02–303.9)	**<0.001**	**<0.001**
**Ox-LDL (ng/mL)**	1643 (1233–1910)	1623 (1066–2154)	1187 (919.5–1887)	**<0.05**	**<0.00001**
**Trx (ng/mL)**	3.90 (2.66–6.88)	4.59 (2.18–11.6)	5.1 (2.7–10.2)	0.38	**<0.05**
**E-selectin (pg/mL)**	5736 (1043–14,414)	5649 (2040–9500)	3828 (717.4–7692)	0.54	**<0.001**
**P-selectin (pg/mL)**	83,493 (35,819–147,352)	95,089 (37,656–188,437)	91,296 (41,620–12,1511)	0.20	0.37
**ICAM-1 (pg/mL)**	99,269 (44,598–130,792)	102,415 (38,572–146,950)	79,565 (23,092–129,154)	0.79	**<0.05**
**VCAM-1 (ng/mL)**	953.8 (753.1–1258)	1001 (761–1394)	838.1 (532–993.7)	0.62	**=0.05**
**OGG-1 (ng/mL)**	4.14 (2.53–5.7)	5.42 (2.4–8.63)	6.8 (3.7–12.8)	0.65	**<0.05**
**Cholesterol (mg/dL)**	201 (114–302)	199 (114–295)	200.4 (114–302)	0.69	0.13
**LDL (mg/dL)**	126 (65–219)	131 (58–216)	127 (65–219)	0.20	0.49
**HDL (mg/dL)**	43 (24–85)	50 (27–80)	46.6 (24–79)	0.20	0.09
**TGs (mg/dL)**	120 (51–684)	120.5 (45–326)	153.3 (51–684)	0.67	0.35
**Vitamin D (ng/mL)**	34.2 (9.6–70.7)	33.0 (9.5–54.2)	38.6 (17.3–57.7)	0.95	0.21
**Glucose (mg/dL)**	89 (80–180)	86 (76–147)	85 (74–145)	0.69	0.69

Abbreviations: **IGF-1**: insulin like growth factor type 1; **Ox-LDL**: oxidized low-density lipoprotein, **Trx**: thioredoxin; **VCAM-1**: vascular cell adhesion molecule 1; **ICAM-1**: intercellular adhesion molecule 1; **OGG-1**: 8-oxoguanine DNA glycosylase 1; **LDL**: low-density lipoprotein; **HDL**: high-density lipoprotein; **TGs**: triglycerides. Values are expressed as median (min–max); *p* value (0 vs. 12 months) from comparison of baseline (V0) and 12-month follow-up using appropriate non-parametric tests; *p* value (0 vs. 24 months) from comparison of baseline (V0) and 24-month follow-up using appropriate non-parametric tests. Baseline and 12-month data partially overlap with previously published results [5].

**Table 2:** The investigated bioimpedance parameters in the studied group.

Parameter					
	Initially	After 12 months	After 24 months	*p* Value (0 vs. 12 months)	*p* Value (0 vs. 24 months)
**Total mass (kg)**	78.6 (39.6–167.3)	78.0 (62.3–156)	86.9 (59.8–167.3)	0.51	**0.05**
**Tissue fat %**	37.5 (27.4–50.4)	38.4 (26.7–48.7)	39.7 (27.4–60.4)	**0.04**	**0.001**
**Fat tissue (g)**	28,434 (13,891–82,462)	29,937 (16,939–67,385)	33,868 (16,881–82,462)	0.08	**0.05**
**Lean mass (g)**	48,646 (23,996–81,228)	45,550 (36,530–84,977)	50,021 (34,296–81,228)	0.49	**0.0008**
**BMC**	2547 (1261–3778)	2568 (1770–3650)	2731 (1844–3778)	0.42	0.72
**L1–L4 BMD**	1.09 (0.8–1.6)	1.1 (0.9–1.5)	1.2 (0.9–1.6)	0.73	**0.04**
**L1–L4 T score**	−1.1 (−3.4–+3.2)	−0.3 (−2.0–+2.4)	−0.1 (−2.5–+3.2)	0.17	0.1
**L1–L4 Z score**	−1.1 (−3.7–+3.0)	−0.9 (−2.3–+2.0)	−0.3 (−2.2–+3.0)	0.73	0.13
**Neck BMD**	0.95 (0.7–1.4)	0.96 (0.77–1.5)	1.04 (0.74–1.4)	0.29	**0.0002**
**Neck T score**	−0.8 (−2.1–+2.3)	−0.6 (−1.9–+2.5)	−0.63 (−2.1–+2.3)	0.79	0.92
**Neck Z score**	−0.9 (−2.2–+2.0)	−1.0 (−2.0–+2.3)	−0.43 (−2.1–+2.0)	0.72	**0.0018**

Abbreviations: **BMC**: bone mineral content; **BMD**: bone mineral density; **“−”:** minus. Values are expressed as median (min–max); *p* value (0 vs. 12 months) from comparison of baseline (V0) and 12-month follow-up using appropriate non-parametric tests; *p* value (0 vs. 24 months) from comparison of baseline (V0) and 24-month follow-up using appropriate non-parametric tests. Baseline and 12-month data partially overlap with previously published results [5].

**Table 3:** Spearman’s correlation coefficients between parameters and other metabolic markers in group at baseline and during the treatment.

Parameter	Initially	After 12 Months	After 24 Months
**Ox-LDL (ng/mL) vs. P-selectin (pg/mL)**	NS	NS	*p* = 0.02 R = 0.60
**Ox-LDL (ng/mL) vs. Lean (g)**	NS	*p* = 0.02 R = 0.63	NS
**E-selectin (pg/mL) vs. VCAM-1 (ng/mL)**	NS	NS	*p* = 0.05 R = 0.57
**E-selectin (pg/mL) vs. HDL (mg/dL)**	NS	NS	*p* = 0.01 R = −0.67
**E-selectin (pg/mL) vs. Waist (cm)**	NS	NS	*p* = 0.04 R = 0.57
**Trx (ng/mL) vs. Tissue (g)**	NS	NS	*p* = 0.04 R = 0.71
**Trx (ng/mL) vs. Vitamin D (mg/dL)**	NS	NS	*p* = 0.01 R = −0.69
**Trx (ng/mL) vs. Fat (g)**	NS	NS	*p* = 0.01 R = 0.71
**ICAM-1 (pg/mL) vs. BMI (kg/m^2^)**	NS	NS	*p* = 0.04 R = 0.55
**ICAM-1 (pg/mL) vs. Waist (cm)**	NS	*p* = 0.03 R = 0.58	*p* = 0.04 R = 0.57
**ICAM-1 (pg/mL) vs. Hip (cm)**	NS	NS	*p* = 0.02 R = 0.61
**ICAM-1 (pg/mL) vs. LDL (mg/L)**	NS	NS	*p* = 0.04 R = −0.57
**P-selectin (pg/mL) vs. BMI (kg/m^2^)**	NS	NS	*p* = 0.02 R = 0.64
**P-selectin (pg/mL) vs. Tissue (g)**	NS	NS	*p* = 0.03 R = 0.63
**P-selectin (pg/mL) vs. Fat (g)**	NS	*p* = 0.04 R = 0.57	NS
**VCAM-1 (ng/mL) vs. Glucose (mg/dL)**	NS	NS	*p* = 0.03 R = 0.58
**LDL (mg/dL) vs. L1–L4 Z-score**	NS	NS	*p* = 0.03 R = −0.58
**LDL (mg/dL) vs. Lean (g)**	NS	NS	*p* = 0.01 R = −0.33
**HDL (mg/dL) vs. IGF-1 (ng/mL)**	NS	NS	*p* = 0.02 R = −0.59
**HDL (mg/dL) vs. Lean (g)**	NS	*p* < 0.01 R = −0.73	*p* = 0.01 R = −0.68
**TGs (mg/dL) vs. L1–L4 T-score**	NS	NS	*p* = 0.03 R = 0.60
**TGs (mg/dL) vs. L1–L4 Z-score**	NS	NS	*p* = 0.05 R = 0.54
**Lean (g) vs. L1–L4 BMD**	NS	*p* < 0.001 R = 0.78	*p* = 0.05 R = 0.55
**Lean (g) vs. L1–L4 T-score**	NS	*p* = 0.01 R = 0.67	NS
**Lean (g) vs. L1–L4 Z-score**	NS	*p* = 0.04 R = 0.53	NS
**Lean (g) vs. Neck BMD**	NS	*p* < 0.01 R = 0.72	*p* = 0.01 R = 0.69
**Lean (g) vs. Neck T-score**	NS	*p* = 0.02 R = 0.68	*p* = 0.03 R = 0.60
**Lean (g) vs. Neck Z-score**	NS	*p* = 0.01 R = 0.67	NS

Abbreviations: **IGF-1**: insulin like growth factor type 1; **Ox-LDL**: oxidized low-density lipoprotein, **Trx**; thioredoxin; **VCAM-1**: vascular cell adhesion molecule 1; **ICAM-1**: intercellular adhesion molecule 1; **OGG-1**: 8-oxoguanine DNA glycosylase 1; **LDL**: low-density lipoprotein; **HDL**: high-density lipoprotein; **TGs**: triglycerides; **BMC**: bone mineral content; **BMD**: bone mineral density; **“−”:** minus; **BMI**: body mass index. Baseline and 12-month data partially overlap with previously published results [5]. Correlations were assessed using Spearman’s rank correlation test.

**Table 4:** Characteristics of the study group [5].

	Sex	Age (Years)	Treatment (Before rhGH)	Dose of rhGH	Etiology GHD	IGF-1 (ng/mL) Initially	BMI (kg/m^2^) Initially	CO-GHD in History
**P1**	F	41	HCT, L, D, Es/Pg	0.5 mg	CPGP	68.6	30.9	+
**P2**	M	25	L, T	0.5 mg	NFPM	62.8	24.8	+
**P3**	M	18	T	0.4 mg	CPH	27.3	22.8	+
**P4**	F	26	HCT, L, Es/Pg	0.6 mg	CPH	40.1	29.0	+
**P5**	M	19	D, L, T, HCT	0.3 mg	CPGP	74.8	34.9	+
**P6**	F	60	HCT, L	0.4 mg	ES	15.11	24.3	-
**P7**	M	20	L, HCT, T	0.3 mg	CPH	91.8	28.1	+
**P8**	M	23	-	0.3 mg	I	138.8	25.9	-
**P9**	F	38	L, HCT, Es/Pg	0.5 mg	NFPM	47.07	24.9	-
**P10**	M	18	T	0.2 mg	I	120.2	20.4	+
**P11**	M	28	L, HCT, T, D	0.3 mg	CPGP	22.6	27.1	+
**P12**	M	42	L, T, D	0.3 mg	CPGP	63.0	54.1	+
**P13**	M	36	HCT, L, T	0.5 mg	CPGP	48.9	21.5	+
**P14**	M	18	L, HCT, D, T	0.7 mg	CPGP	54.4	24.4	+
**P15**	M	25	L, HCT, T	0.5 mg	CPGP	8.6	35.8	+

Abbreviations: **GHD**: growth hormone deficiency; **rhGH**: recombinant human growth hormone; **P**: patient; **F**: female; **M**: male; **HCT**: hydrocortisone; **L**: levothyroxine; **Es/Pg**: estrogen/progesterone; **D**: desmopressin; **T**: testosterone; **CPH**: congenital pituitary hypoplasia; **CPGP**: craniopharyngioma postsurgical; **ES**: empty sella; **NFPM**: non-functioning pituitary macroadenoma; **CO-GHD:** childhood-onset growth hormone deficiency; **I**: idiopathic; **IGF-1**: insulin-like growth factor type 1; **BMI**: body mass index.

## Data Availability

The original contributions presented in this study are included in the article. Further inquiries can be directed to the corresponding author.

## Abbreviations

IGF-1, insulin-like growth factor 1; Ox-LDL, oxidized low-density lipoprotein; Trx, thioredoxin; VCAM-1, vascular cell adhesion molecule 1; ICAM-1, intercellular adhesion molecule 1; OGG-1, 8-oxoguanine DNA glycosylase 1; LDL, low-density lipoprotein; HDL, high-density lipoprotein; TGs, triglycerides; BMC, bone mineral content; BMD, bone mineral density; BMI, body mass index; GHD, growth hormone deficiency; rhGH, recombinant human growth hormone; P, patient; F, female; M, male; HCT, hydrocortisone; L, levothyroxine; Es/Pg, estrogen/progesterone; D, desmopressin; T, testosterone; CPH, congenital pituitary hypoplasia; CPGP, craniopharyngioma postsurgical; ES, empty sella; NFPM, non-functioning pituitary macroadenoma; CO-GHD, childhood-onset growth hormone deficiency; I, idiopathic; “−”, minus.
